# Green Tea Attenuates the Particulate Matter (PM)_2.5_-Exposed Gut-Brain Axis Dysfunction through Regulation of Intestinal Microenvironment and Hormonal Changes

**DOI:** 10.4014/jmb.2409.09035

**Published:** 2024-10-31

**Authors:** Jong Min Kim, Hyo Lim Lee, Min Ji Go, Hyun-Jin Kim, Mi Jeong Sung, Ho Jin Heo

**Affiliations:** 1 Division of Applied Life Science (BK21), Institute of Agriculture and Life Science, Gyeongsang National University, Jinju 52828, Republic of Korea; 2 Korea Food Research Institute, Wanju-gun 55365, Republic of Korea

**Keywords:** Matcha, particulate matter, intestinal microenvironment, gut-brain axis, hormonal changes, brain metabolites

## Abstract

Chronic exposure to particulate matter (PM)_2.5_ causes brain damage through intestinal imbalance. This study was estimated to confirm the regulatory activity of green tea against chronic PM_2.5_ exposure-induced abnormal gut-brain axis (GBA) in BALB/c mice. The green tea, as an aqueous extract of matcha (EM), ameliorated the colon length, short chain fatty acid contents, antioxidant biomarkers, myeloperoxidase (MPO) activity, and serum inflammatory cytokines. EM regulated the gut microbiota related to tryptophan intake and hormone metabolism. EM showed regulatory effect of intestinal tight junction (TJ) protein, inflammatory response, and apoptotic biomarkers. In addition, EM improved PM_2.5_-induced tryptophan-related hormonal metabolic dysfunction in intestinal tissue and serum. Through the ameliorating effect on GBA function, the consumption of EM presented the protective effect against inflammatory effect, apoptosis, synaptic damage, and hormonal activity in cerebral tissue, and suppressed abnormal change of brain lipid metabolites. In particular, EM intake showed relatively excellent improvement effects on indicators including *Bacteroides*, *Ruminococcus*, *Murinobaculaceae*, *Allopreyotella*, cyclooxygenase-2 (COX-2), acetylcholinesterase (AChE), 11,12-dihydroxyeicosatrienoic acid (DHET), and intestinal acetate from the PM group. These findings indicate that the dietary intake of EM might provide a regulatory effect against PM_2.5_-exposed GBA dysfunction via the intestinal microbiota and hormonal changes.

## Introduction

In urban environments, air pollution including micro-, and nanoparticles is reported to affect human health through respiratory dysfunction, cardiovascular disease, type 2 diabetes, and systemic inflammation. This air pollution is composed of a complex mixture of particulate matter (PM) containing polycyclic aromatic hydrocarbons, heavy metals, organic chemicals, and bacteria [1]. In particular, ambient PM_2.5_ (aerodynamic diameter ≤ 2.5 μm) absorbed through various pathways causes detrimental toxicity in the human body [2]. PM_2.5_ causes systemic inflammation by promoting the secretion of cytokines such as interleukin (IL)-1β and tumor necrosis factor-α (TNF-α) [3]. While previous studies of PM mainly focused on respiratory, dermal, and cardiovascular disease, various studies related to the impact of air pollution on the gastrointestinal (GI) tract have also been reported [4].

PM_2.5_ can easily enter through the GI tract and causes epithelial permeability, immune barrier function damage, and gut microbiome change [5]. Notably, the GI tract plays an important role in regulating the digestion and absorption of food and nutrients [6], while the intestinal barrier can be easily affected by microenvironment changes such as inflammatory reaction, absorption of external materials, and dysbiosis [7]. In particular, PM_2.5_ damages the epithelial cells that divide the intestinal environment and the circulatory system, thereby disrupting the intestinal barrier and increasing permeability [8]. These impairments in gut function and metabolic disorders affect the brain through the gut-brain axis (GBA), the communication network between the gut and the brain [9]. The impact of PM_2.5_ on GBA occurs through multiple pathways. PM_2.5_ exposure induces oxidative stress and inflammation through gut microbiota imbalance, further weakening the gut barrier and allowing inflammatory cytokines to enter the bloodstream [9]. Disorders of the GBA regulate neurotransmitters and neurogenesis, affect hormone metabolism, and ultimately cause synaptic damage, neuroinflammation, and apoptosis, resulting in damage to brain tissue [2]. Therefore, it is important to regulate the inflammatory response and dysbiosis in gut environments to prevent a PM_2.5_-induced abnormal hormonal pathway and GBA function. Especially, the risk of PM cannot be easily eliminated because it is inevitably exposed directly or indirectly in the general environment [2]. Therefore, it is very important to form a defense ability against PM_2.5_ in advance through the intake of functional foods or materials with excellent physiological activity.

Green tea is widely known to have various physiological activities such as an anti-inflammatory effect, anti-amnesic effect, and anti-diabetic effect derived from large amounts of phenolic compounds and tannins [10, 11]. In particular, green tea contains catechins such as (−)-epicatechin, (−)-epigallocatechin, (−)-epicatechin-3-gallate, and (−)-epigallocatechin-3-gallate (EGCG) with various bioactive effects [12]. In particular, EGCG present in green tea extract has been reported to exhibit anti-inflammatory activity by regulating pathways such as TLR/NF-kB/MAPK in various animal models, and also prevents apoptosis and mitochondrial damage based on its excellent antioxidant activity [13]. In particular, catechins absorbed into the body can penetrate the BBB of brain tissue and be delivered to neuronal cells, and various physiological activities have been reported [14]. To improve the physiological activity, catechin content, and palatability of green tea, it was processed in previous studies to produce a matcha using shade-cultivated and superheated green tea [10]. This green tea improved the metabolic imbalance in high-fat diet-induced diabetic mice by a regulatory effect on hepatic and cerebral dysfunction [15]. In addition, matcha green tea ameliorated PM_2.5_-induced cognitive and memory deficits by regulating systemic inflammation in dermal, pulmonary, and cerebral tissues [16]. However, there are few studies about the intestinal protective effect of green tea and/or green tea products against PM_2.5_-induced GBA dysfunction. Therefore, the regulatory activity of green tea was evaluated by regulation of GBA by microenvironment change in chronic PM_2.5_-exposed BALB/c mice.

## Materials and Methods

### Green Tea Extract Preparation

The green tea sample (Institute of Hadong Green Tea, Republic of Korea) was treated according to Kim *et al*.[10], and extracted with 50-fold distilled water at 40°C for 2h. The extracted green tea sample was evaporated and lyophilized for experimental analysis.

### Animals and Diet

Six-week-old male BALB/c mice were obtained from an animal supplier (Samtako, Republic of Korea). The animals were randomly divided into three/four per cage (12 h light/dark cycle; 55% humidity; and 22 ± 2°C). The in vivo groups were divided into six groups with a non-chamber stress control (Sham) group (non-exposure/vehicle intake), normal control (NC) group (clean air-exposure/vehicle intake), normal sample (NS) group (clean air-exposure/40 mg/kg of body weight (B.W.) intake^+^), PM_2.5_-exposed group (PM air-exposure/vehicle intake), and two PM_2.5_-exposed with extract of match (EM) treatment groups (PM air-exposure/20 and 40 mg/kg of B.W.; EM20 and EM40, respectively). The sample was orally fed for 12 weeks. The sample concentration and period were determined based on the results obtained through previous study [16]. The 500 μg/m^3^ PM dissolved in purified water was dispersed in aerosol form into the chamber for 5 h per day during a designated sample intake period.

### Biological Sample Preparation

The experimental mice were sacrificed by CO_2_ anesthesia, and blood, intestinal, and cerebral tissues were harvested. The intestinal tissues were homogenized with a bullet blender (Next Advance Inc., USA) with phosphate-buffered saline (PBS) or 10 mM phosphate buffer with 1 mM EDTA (pH 6.7) at 4°C. The blood obtained from the postcaval vein centrifuged at 13,000 ×*g* for 10 min and the supernatant sample were used for analysis for inflammatory markers and hormonal changes. To analyze fecal analysis for fatty acid contents and gut microbiome, fecal samples were obtained by gently rubbing the abdomen of the mice, prompting natural defecation to maintain sample integrity.

### Short Chain Fatty Acids (SCFAs) in Feces Contents

The feces homogenized in 5 mM NaOH were centrifuged at 12,000 ×*g* for 10 min. The supernatant was added into propanol/pyridine (v/v = 3:2) and propyl chloroformate. This mixture dissolved in hexane were re-centrifuged at 15,000 ×*g* for 10 min. The hexane layer was used for the evaluation of SCFAs contents using an Agilent 7890A Gas Chromatograph (Agilent, USA). The conditions were performed according to Kim *et al*. [10].

### Antioxidant Biomarkers

To evaluate the malondialdehyde (MDA) contents, the intestinal tissues homogenized with PBS were centrifuged at 2,500 ×*g* for 10 min at 4°C. The supernatants were reacted with 1% phosphoric acid and 0.67% thiobarbituric acid at 95°C for 1 h. The absorbance was evaluated at 532 nm (Epoch 2, BioTek Instruments Inc., USA) [10].

To evaluate the reduced glutathione (GSH) contents, the intestinal tissues homogenized with phosphate buffer (pH6.0) were centrifuged at 10,000 ×*g* for 15 min at 4°C. The supernatants were reacted with 5% metaphosphoric acid and re-centrifuged at 2,000 ×*g* to remove large proteins. The supernatants were mixed with 0.26 M tris-HCl (pH 7.8), 0.65 N NaOH, and 1 mg/mL of o-phthaldialdehyde at 37°C for 15 min. The fluorescence was assessed at 320 nm (excitation) and 420 nm (emission) using a fluorescence reader (Infinite 200, Tecan Co., Switzerland)[17].

To evaluate the superoxide dismutase (SOD) activity, the intestinal tissues homogenized with PBS were centrifuged at 400 ×*g* for 10 min at 4°C. The pellets were extracted in extraction buffer (10% SOD buffer, 0.4% (v/v) triton X-100, and 200 μM phenylmethane sulfonylfluoride), and centrifuged at 10,000 ×*g* for 10 min at 4°C. The SOD activity was using a SOD analysis kit (Dojindo Molecular Technologies).

To evaluate the myeloperoxidase (MPO) activity, the intestinal tissues homogenized with 0.5% hexade-cyltrimethylammonium bromide in 50 mM phosphate buffer (pH 6.0) were centrifuged at 15,000 ×*g* for 15 min at 4°C. The supernatants were reacted with 50 mM potassium phosphate buffer (pH 6.0) with 5.26 mM *o*-dianisidine dihydrochloride and 0.0005% H_2_O_2_. The absorbance of reactant was detected at 450 nm (BioTek Instruments Inc.)[10].

### Inflammatory Cytokines

To evaluate the inflammatory cytokines in serum, the TNF-α (MTA00B, R&D Systems, NE USA), IL-1β (MLB00C, R&D Systems), IL-6 (M6000B, R&D Systems), and interferon-γ (IFN-γ) (MIF00, R&D Systems) levels were measured using commercial kits according to the manufacture's protocols.

### 16s rRNA Gene Amplification

PCR was started immediately after the DNA was extracted. The 16S rRNA V3-V4 amplicon was amplified using KAPA HIFI Hot Start Ready Mix (2×) (Roche, Switzerland). Universal bacterial 16S rRNA gene amplicon PCR primers including Illumina overhang adapter sequences were used: the forward primer was (341F: 5’-TCGTCGGCAGCGTCAGATGTGTATAAGAGACAGCCTACGGGNGGCWGCAG-3’) and reverse primer (806R: 5’-GTCTCGTGGGCTCGGAGATGTGTATAAGAGACAGGACTACHVGGGTATCTAATCC-3’) (Illumina, USA).

### 16S Gene Library Construction, Quantification, and Sequencing

Purify PCR amplicons away from free primers and primer dimers. To sequence this amplicon, those were attached with dual indices and Illumina sequencing adapters using the Nextera XT Index Kit (Illumina), and the amplicon was purified again using AMPure XP beads. Before sequencing, the DNA concentration of each PCR product was determined using a Qubit 3.0 Fluorometer (Thermo Fisher Scientific, USA), and it was quality controlled using a bioanalyzer (Agilent 2100, Agilent Technologies, Inc., USA). Sequencing was performed using the Illumina MiSeq system (Illumina MiSeq, Illumina).

### Western Blot Analysis for Protein Expression

The intestinal and cerebral tissues were homogenized with a lysis buffer (GeneAll Biotechnology, Republic of Korea) with 1% protease inhibitor. The extraction was centrifuged at 13,000 ×*g* for 10min at 4°C and these supernatants were used for protein analysis. The extracted samples were separated through the SDS-PAGE gel and electrotransferred to a polyvinylidene fluoride membrane. The transferred membranes were reacted in primary antibodies at 4°C for 12 h. Then the membranes were reacted with secondary antibodies for 1 h at 25°C. The reactants were detected using an image analyzer (iBright Imager, Thermo Fisher Scientific). The densities of expression level were calculated using an analytical software (ImageJ, National Institutes of Health, USA). Antibody details are showed in Table S1.

### Metabolite Changes

Hormonal and cerebral metabolites were analyzed with an UPLC system (Waters Corp., USA). The samples were injected into an Acquity UPLC BEH C_18_ column (2.1 mm × 100 mm, 1.7 μm; Waters Corp.). The metabolites were analyzed using UPLC Q-TOF MS^E^ (Waters Corp.) with positive electrospray ionization (ESI) positive mode. The MRM conditions for the analytes are shown in Table S2.

### Statistical Analysis

All experimental results were expressed as mean ± standard deviation (SD). Statistically significant differences were presented as one-way analysis of variance (ANOVA) and identified using Duncan’s new multirange test (*p* < 0.05) with the SAS program (version 9.4, SAS Institute Inc., USA).

## Results

### Change of Colon Length

**Fig. 1:**
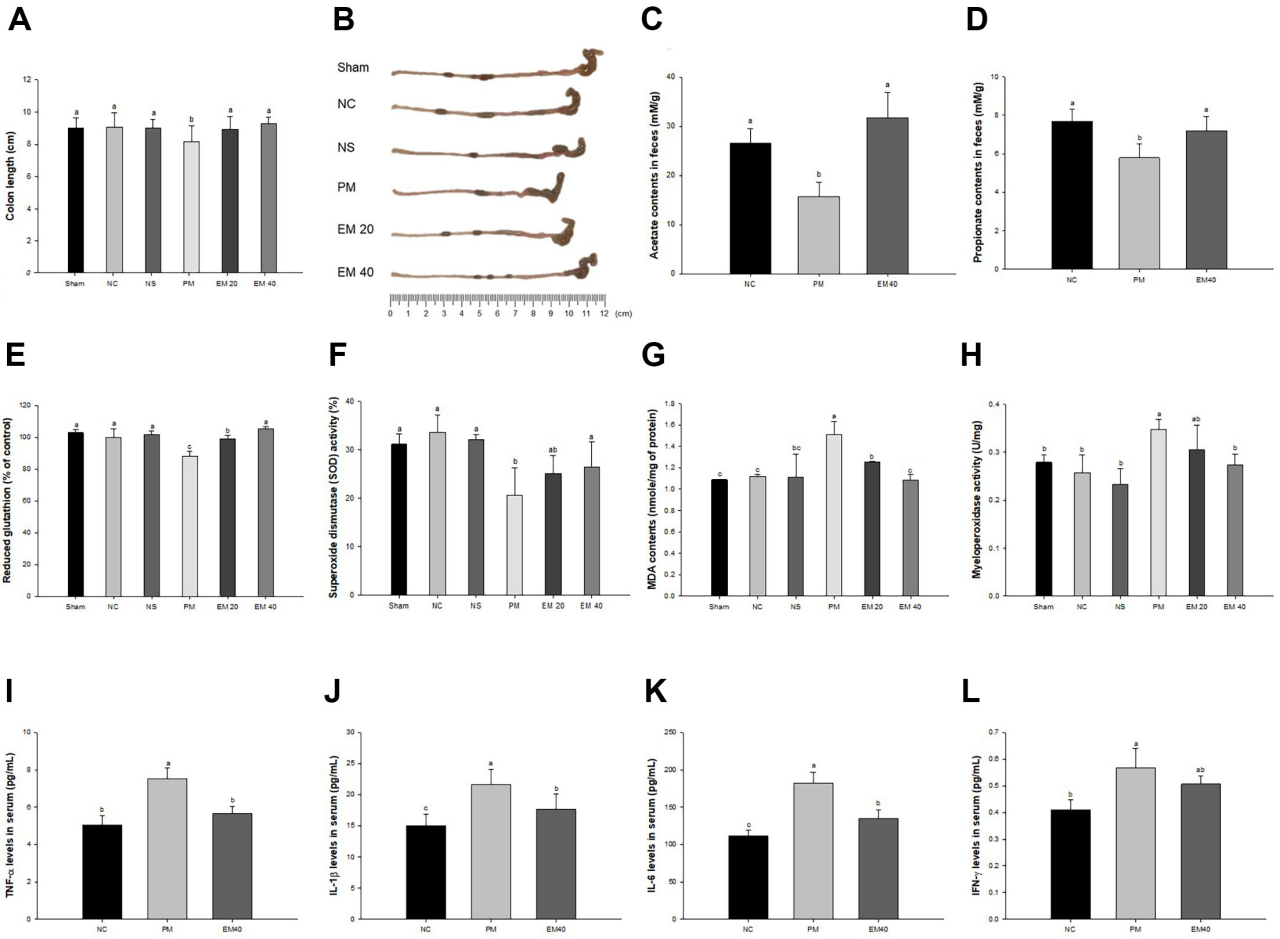
Protective effect of aqueous extract of matcha (EM) on colon length (A) relative colon image (B) fecal acetate content (C) fecal propionate content (D) reduced GSH content (E) SOD content (F) MDA content (G) and MPO activity (H) in intestinal tissue, TNF-α level (I) IL-1β level (J) IL-6 level (K) and IFN-γ level (L) in serum. Results shown are mean ± SD (*n* = 5). Data were considered statistically significant at *p* < 0.05 and different small letters represent statistical differences.

Colon length and a representative image are shown in Fig. 1A and 1B. The colon length of the Sham (9.02 cm), NC (9.06 cm) and NS (9.00 cm) groups confirmed no significant differences. The colon length of the PM group (8.16 cm) decreased more than that of the NC group. While, that of the EM group (EM20, 8.94 cm; EM40, 9.26 cm) was improved more than the PM group.

### Fecal SCFAs Contents

Fecal SCFAs concentrations are shown in Fig. 1C and 1D. The SCFAs concentration of the PM group (acetate, 15.70 mM/g; propionate, 5.78 mM/g) was downregulated compared to the NC group (acetate, 26.52 mM/g; mM/g; propionate, 7.67 mM/g). The EM40 group (acetate, 31.74 mM/g; mM/g; propionate, 7.18 mM/g) improved the fecal SCFAs concentration.

### Antioxidant Biomarkers

Reduced intestinal GSH levels are shown in Fig. 1E and 1F. The reduced GSH level and SOD activity of the Sham (102.88% and 31.16%, respectively), NC (100.00% and 33.65%, respectively), and NS (101.81% and 32.11%, respectively) groups confirmed no significant differences. The GSH level of the PM group (88.19% and 20.64%, respectively) was reduced more than that of the NC group. However, that of the EM group (EM20, 99.08% and 25.17%; EM40, 105.37% and 26.46%, respectively) improved more than the PM group.

Intestinal MDA contents are shown in Fig. 1G and 1H. The MDA content and MPO activity of the Sham (1.09 nmole/mg of protein and 0.28 U/mg, respectively), NC (1.12 nmole/mg of protein and 0.26 U/mg, respectively) and NS (1.11 nmole/mg of protein and 0.23 U/mg, respectively) groups confirmed no significant differences. The MDA content of the PM group (1.51 nmole/mg of protein and 0.35 U/mg, respectively) decreased more than the that of the NC group. However, that of the EM group (EM20, 1.25 nmole/mg of protein and 0.30 U/mg; EM40, 1.08 nmole/mg of protein and 0.27 U/mg, respectively) improved more than the PM group.

### Serum Inflammatory Cytokines

Serum cytokines levels are shown in Fig. 1I-1L. The TNF-α, IL-1β, IL-6, and INF-γ levels of the PM group (7.51, 21.60, 182.17, and 0.57 pg/ml, respectively) increased more than that of the NC group (5.04, 15.03, 111.38, and 0.41 pg/ml, respectively). However, that of the EM group (5.67, 17.72, 137.92, and 0.51 pg/ml, respectively) was suppressed more than the PM group.

### Gut Microbiome

**Fig. 2:**
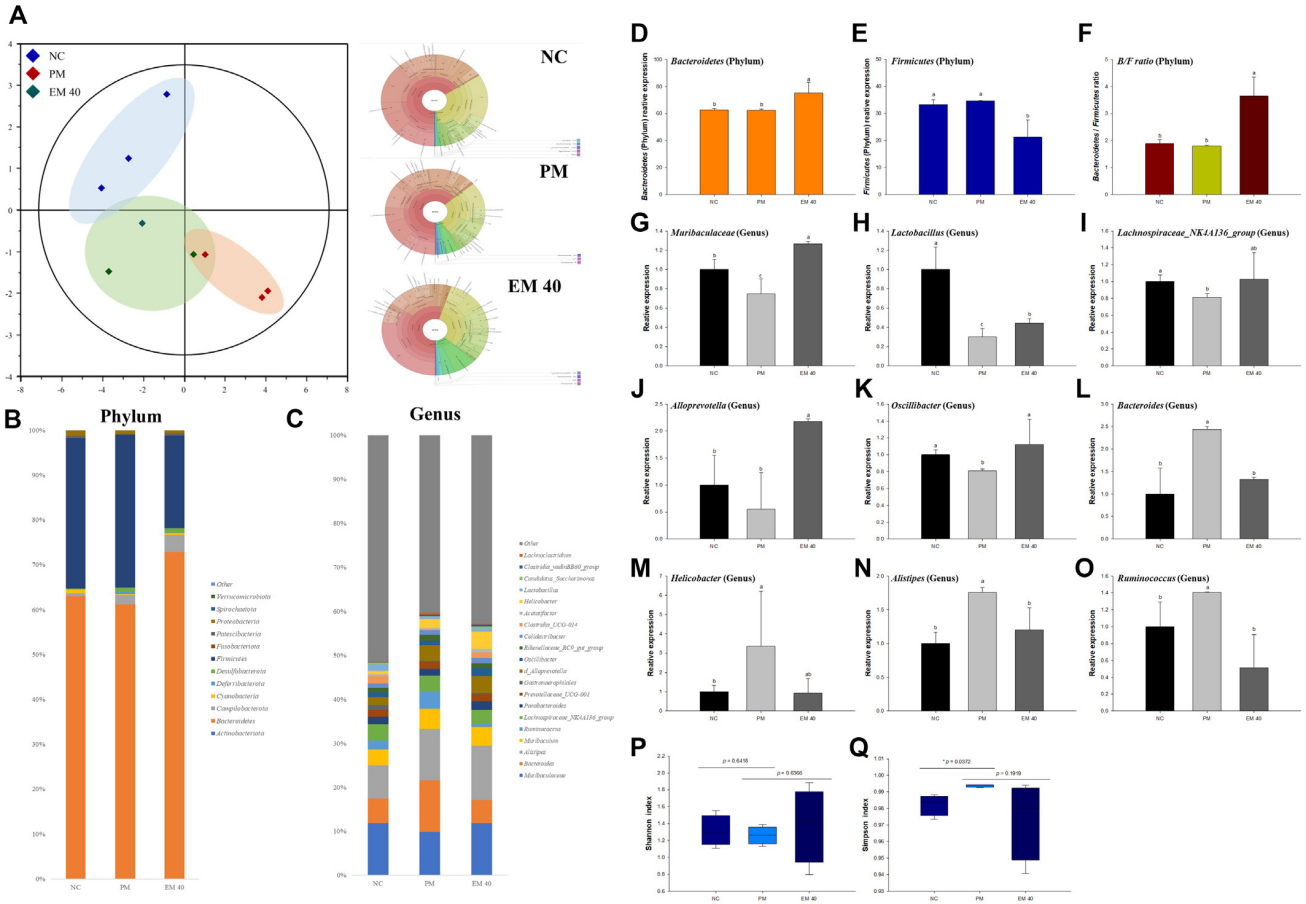
Relative abundance (%) of the gut microbiome at the genera level by aqueous extract of matcha (EM) in PM_2.5_-induced gut dysbiosis. Krona plot of identified bacteria (**A**) relative abundances of the Phylum (**B**) and Genus (**C**) in each group, changes in fecal genera (**D-O**) Shannon index (**P**) and Simpson index (**Q**). Results shown are mean ± SD (*n* = 3). Data were considered statistically significant at *p* < 0.05 and different small letters represent statistical differences.

Gut microbiota changes are presented in Fig. 2. A representative Krona plot of the identified bacteria of each group is shown in Fig. 2A. The relative abundances of the phylum of the NC group (Bacteroidetes, 62.52%; *Firmicutes*, 33.19%; *Bacteroidetes*/Firmicutes ratio, 1.89) and PM group (Bacteroidetes, 62.22%; *Firmicutes*, 34.65%; *Bacteroidetes*/Firmicutes ratio, 1.80) confirmed no significant differences (Fig. 2B), whereas the relative abundances of the phylum of the NS group (Bacteroidetes, 75.21%; *Firmicutes*, 21.25%; *Bacteroidetes*/Firmicutes ratio, 3.64) were different. The relative abundances of the genus are presented in Fig. 2C. The relative differences in fecal genera are presented in Fig. 2D-2Q. The relative abundances of *Muribaculaceae* (0.74), *Lactobacillus* (0.30), *Lachnospiraceae*_NK4A136_group (0.81), *Alloprevotella* (0.55), *Oscillibacter* (0.81), *Bacteroides* (2.44), *Helicobacter* (3.36), *Alistipes* (1.76), and *Ruminococcus* (1.40) of the PM group were different compared to those of the NC group. On the other hand, those of the EM40 group (*Muribaculaceae* (1.26), *Lactobacillus* (0.44), *Lachnospiraceae*_NK4A136_group (1.03), *Alloprevotella* (2.18), *Oscillibacter* (1.12), *Bacteroides* (1.32), *Helicobacter* (1.12), *Alistipes* (1.20), and *Ruminococcus* (0.52) were different.

### Intestinal Protein Expression Level

**Fig. 3:**
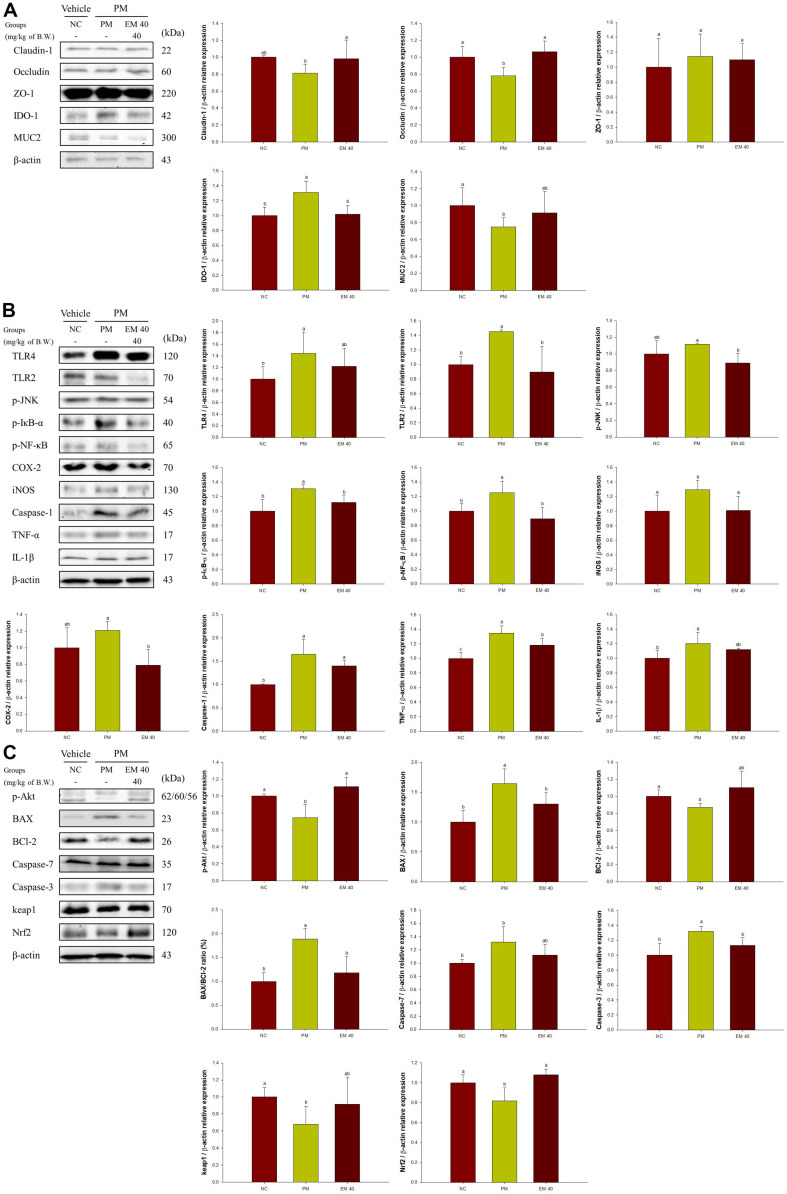
Effect of aqueous extract of matcha (EM) on protein expression of Western blot images. Tight junction protein expression levels of claudin-1, occludin, ZO-1, IDO-1, and MUC2 (A), inflammatory protein expression levels of TLR4, TLR2 p-JNK, p-IκB-α, p-NF-κB, COX-2, iNOS, caspase-1, TNF-α, and IL-1β (B) and cytotoxic protein expression levels of p-Akt, BAX, BCl-2, BAX/BCl-2 ratio, caspase-7, caspase-3, keap1, and Nrf2 (C) in intestinal tissues. Results shown are mean ± SD (*n* = 3). Data were considered statistically significant at *p* < 0.05 and different small letters represent statistical differences.

Intestinal protein expressions related to TJ are presented in Fig. 3A. Claudin-1 (81.10%), occludin (78.08%), and mucin 2 (MUC2) (74.81%) expression levels of the PM group were reduced compared to the NC group. The EM40 group statistically improved claudin-1 (98.07%), occludin (106.48%), and MUC2 (91.47%) levels. IDO-1 (131.18%) expression levels of the PM group were increased compared to the NC group. The EM40 group down-regulated indoleamine 2,3-dioxygenase 1 (IDO-1) (102.15%) levels. The expression of zonula occludens-1 (ZO-1) presented no significant difference between all groups.

Intestinal protein expressions related to inflammation are presented in Fig. 3B. Toll-like receptor (TLR)4 (144.51%), TLR2 (145.13%), phosphorylated c-Jun N-terminal kinases (p-JNK) (111.57%), phosphorylated NFKB inhibitor α (p-IκB-α) (130.57%), phosphorylated nuclear factor kappa-light-chain-enhancer of activated B cells (p-NF-κB) (125.20%), cyclooxygenase-2 (COX-2) (120.75%), inducible nitric oxide synthase (iNOS)(129.50%), caspase-1 (164.81%), TNF-α (134.84%), and IL-1β (120.33%) expression levels of the PM group were increased compared to the NC group. However, the EM40 group statistically down-regulated TLR4 (122.01%), TLR2 (89.68%), p-JNK (88.79%), p-IκB-α (112.05%), p-NF-κB (89.17%), COX-2 (79.08%), iNOS (101.05%), caspase-1 (140.08%), TNF-α (118.05%), and IL-1β (111.80%) levels.

Intestinal protein expressions related to cytotoxicity are presented in Fig. 3C. Phosphorylated protein kinase B (p-Akt) (74.61%), B-cell lymphoma 2 (BCl-2) (87.13%), Kelch-like ECH-associated protein 1 (keap1) (68.19%), and nuclear factor erythroid-2-related factor 2 (Nrf2) (81.71%) expression levels of the PM group were reduced compared to the NC group. However, the EM40 group statistically up-regulated p-Akt (111.02%), BCl-2 (110.24%), keap1 (91.71%), and Nrf2 (108.17%) levels. BAX (164.51%), caspase-7 (131.87%), and caspase-3 (132.05%) expression levels of the PM group increased compared to the NC group. However, the EM40 group statistically down-regulated BAX (130.08%), caspase-7 (112.35%), and caspase-3 (113.08%) levels.

### Hormonal Changes

**Fig. 4:**
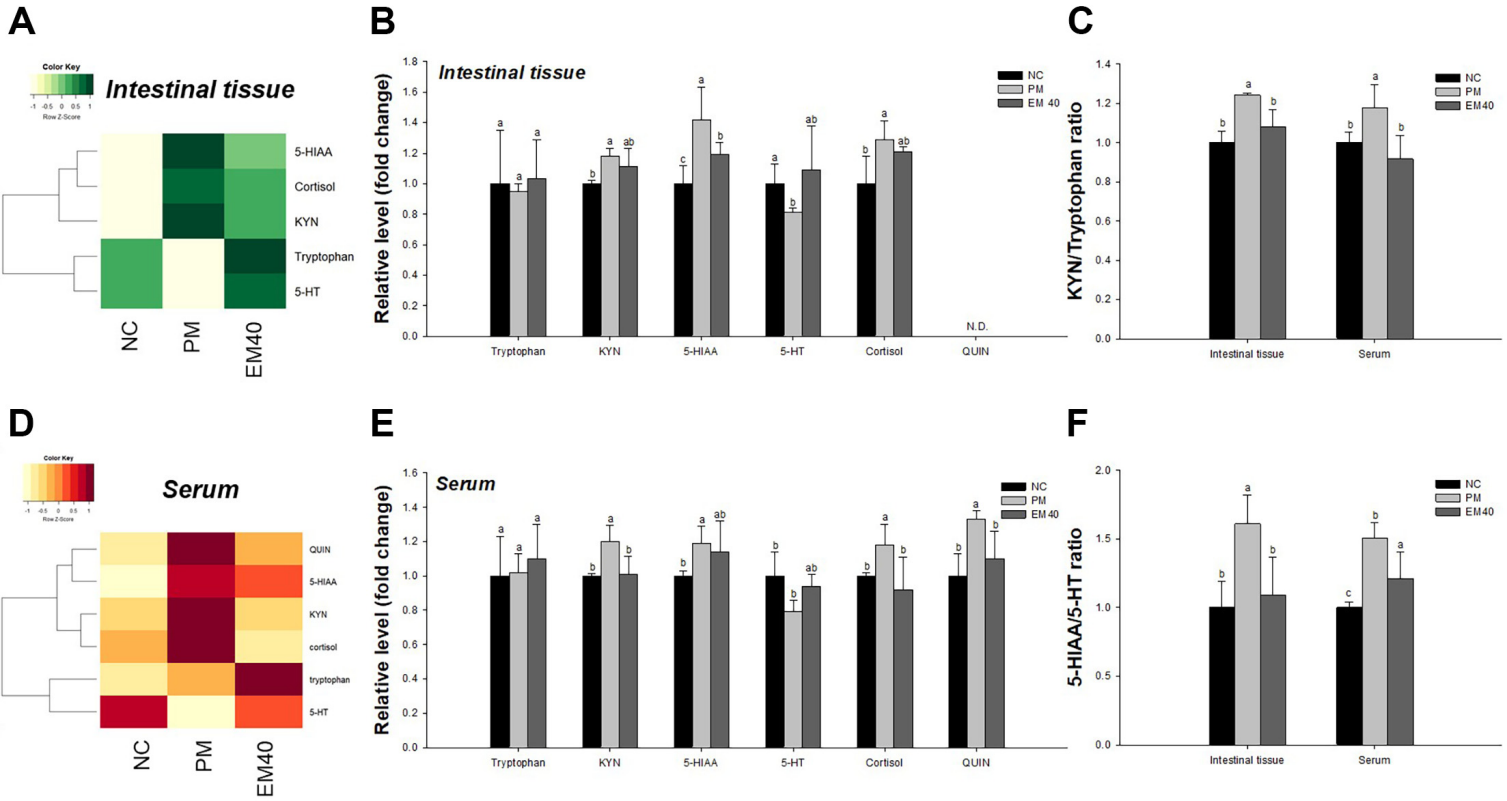
Effect of aqueous extract of matcha (EM) on hormonal changes in intestinal tissue (A-C) and serum (D-F). Results shown are mean ± SD (*n* = 3). Data were considered statistically significant at *p* < 0.05 and different small letters represent statistical differences.

Intestinal and serum hormonal changes are shown in Fig. 4. Intestinal and serum tryptophan levels confirmed no significant difference between all groups. The intestinal 5-hydroxytryptamine (serotonin, 5-HT) (0.88) level of the PM group was reduced compared to that of the NC group. However, the 5-HT (1.09) level of the EM40 group increased. The intestinal 5-hydroxyindoleacetic acid (5-HIAA) (1.42), 5-HIAA/5-HT ratio (1.61), and cortisol (1.29) levels of the PM group were increased compared to those of the NC group. However, the 5-HIAA (1.19), 5-HIAA/5-HT ratio (1.09), and cortisol (1.21) levels of the EM40 group were reduced. The serum 5-HT (0.79) level of the PM group was reduced compared to that of the NC group. However, the 5-HT (0.94) level of the EM40 group increased. The serum quinolinic acid (QUIN) (1.33), 5-HIAA (1.19), 5-HIAA/5-HT ratio (1.51) and cortisol (1.18) levels of the PM group increased compared to those of the NC group. However, the QUIN (1.10), 5-HIAA (1.14), 5-HIAA/5-HT ratio (1.21), and cortisol (0.92) levels of the EM40 group were reduced.

### Cerebral Protein Expression Level

**Fig. 5:**
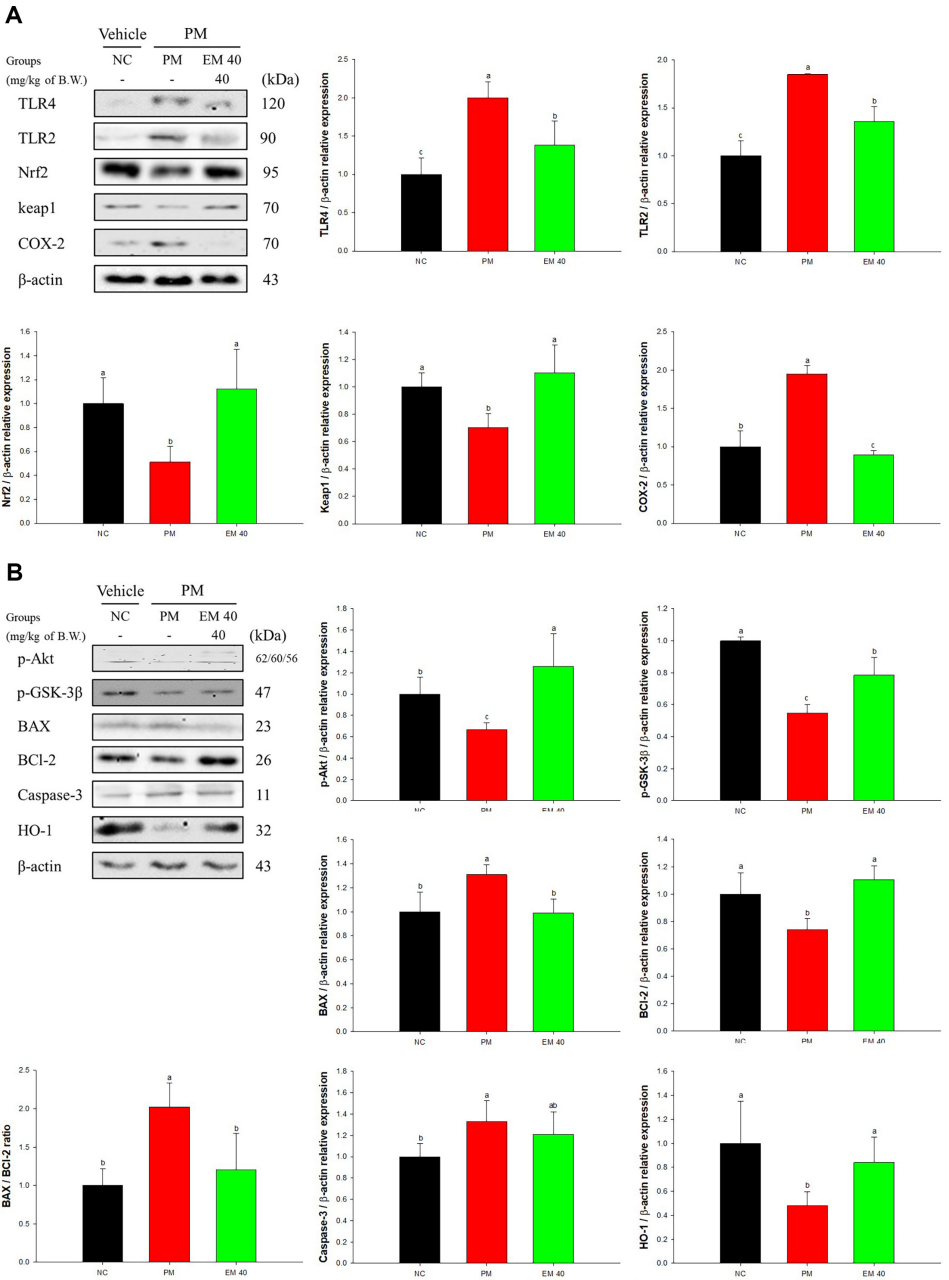
Effect of aqueous extract of matcha (EM) on protein expression of Western blot images. Inflammatory protein expression levels of TLR4, TLR2 Nrf2, keap1, and COX-2 (**A**) and cytotoxic protein expression levels of p-Akt, p-GSK- 3β BAX, BCl-2, BAX/BCl-2 ratio, caspase-3, and HO-1 (**B**) in cerebral tissues. Results shown are mean ± SD (*n* = 3). Data were considered statistically significant at *p* < 0.05 and different small letters represent statistical differences.

Cerebral protein expressions related to inflammation are presented in Fig. 5A. TLR4 (200.15%), TLR2 (184.87%), and COX-2 (195.07%) expression levels of the PM group were increased compared to the NC group. However, the EM40 group statistically down-regulated TLR4 (138.41%), TLR2 (135.97%), and COX-2 (89.07%) levels. Nrf2 (51.18%) and keap1 (70.39%) expression levels of the PM group were reduced compared to the NC group. The EM40 group statistically improved Nrf2 (112.15%) and keap1 (110.51%) levels.

Cerebral protein expressions related to cytotoxicity are presented in Fig. 5B. p-Akt (68.58%), phosphorylated glycogen synthase kinase-3β (p-GSK-3β) (54.81%), BCl-2 (74.19%), and hemeoxiganase-1 (HO-1) (48.17%) expression levels of the PM group decreased compared to the NC group. However, the EM40 group statistically up-regulated p-Akt (125.90%), p-GSK-3β (78.48%), BCl-2 (110.50%), and HO-1 (84.10%) levels. BAX (131.05%) and caspase-3 (133.20%) expression levels of the PM group were increased compared to the NC group. However, the EM40 group statistically down-regulated BAX (99.08%) and caspase-3 (120.97%) levels.

**Fig. 6:**
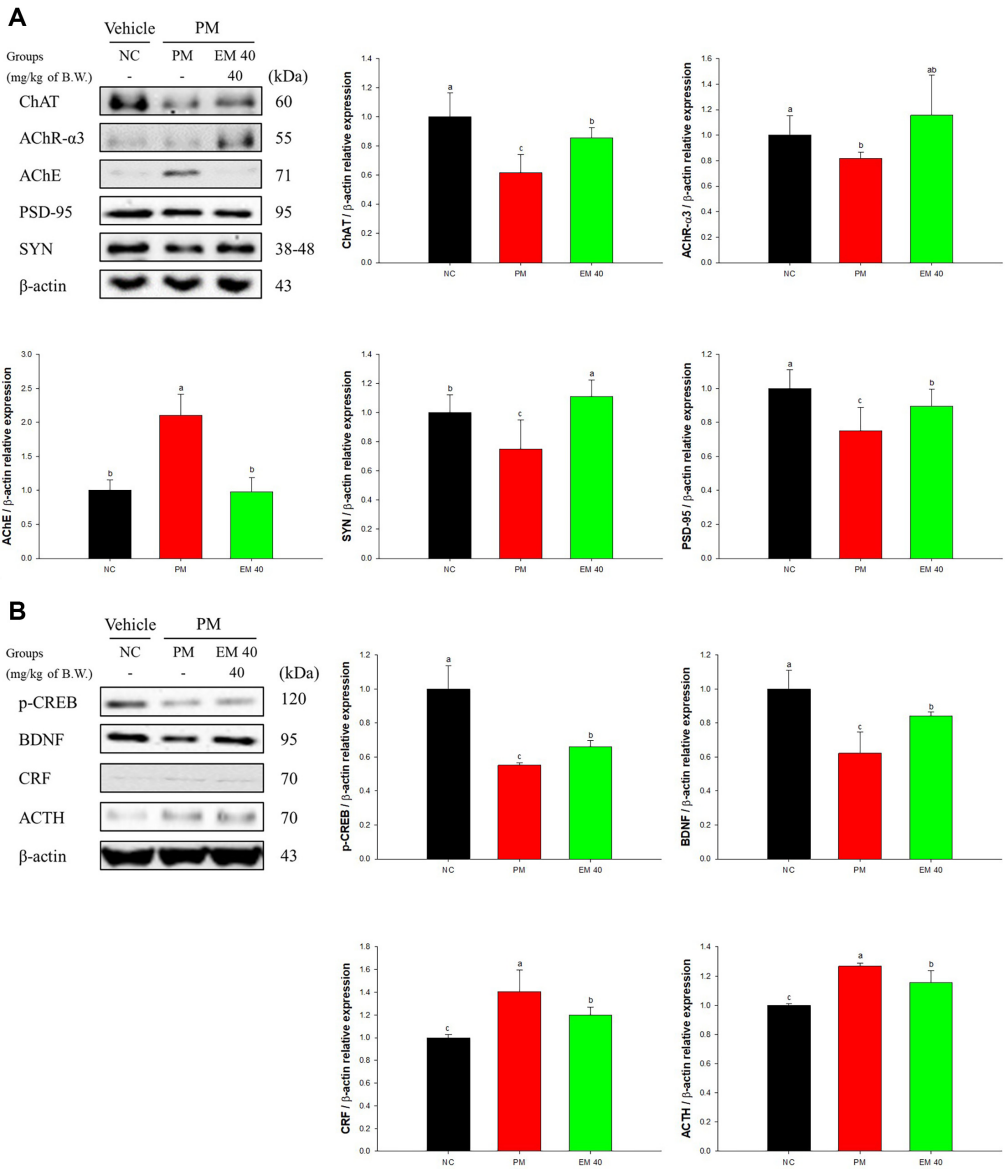
Effect of aqueous extract of matcha (EM) on protein expression of Western blot images. Cholinergic and synaptic protein expression levels of ChAT, AChR-α3 AChE, PSD-95, and SYN (**A**) and hormonal function protein expression levels of p-CREB, BDNF CRF, and ACTH (**B**) in cerebral tissues. Results shown are mean ± SD (*n* = 3). Data were considered statistically significant at *p* < 0.05 and different small letters represent statistical differences.

Cerebral protein expressions related to cholinergic and synaptic function are presented in Fig. 6A. Choline acetyltransferase (ChAT) (61.80%), acetylcholine receptor subunit alpha-3 (AChR-α3) (81.65%), postsynaptic density protein 95 (PSD-95) (75.14%), and synaptophysin (SYN) (75.15%) expression levels of the PM group decreased compared to the NC group. However, the EM40 group up-regulated ChAT (85.61%), AChR-α3 (115.59%), PSD-95 (89.47%), and SYN (111.02%) levels. Acetylcholinesterase (AChE) (210.57%) expression levels of the PM group were increased compared to the NC group. However, the EM40 group statistically down-regulated AChE (98.17%) levels.

Cerebral protein expressions related to hormonal function are presented in Fig. 6B. Phosphorylated cAMP response element-binding protein (p-CREB) (55.17%) and brain-derived neurotrophic factor (BDNF) (62.17%) expression levels of the PM group decreased compared to the NC group. However, the EM40 group statistically up-regulated p-CREB (65.97%) and BDNF (84.17%) levels. Corticotropin releasing factor (CRF) (140.59%) and adrenocorticotropic hormone (ACTH) (126.81%) expression levels of the PM group were increased compared to the NC group. However, the EM40 group statistically down-regulated CRF (120.15%) and ACTH (115.41%) levels.

### Cerebral Metabolite Changes

**Fig. 7:**
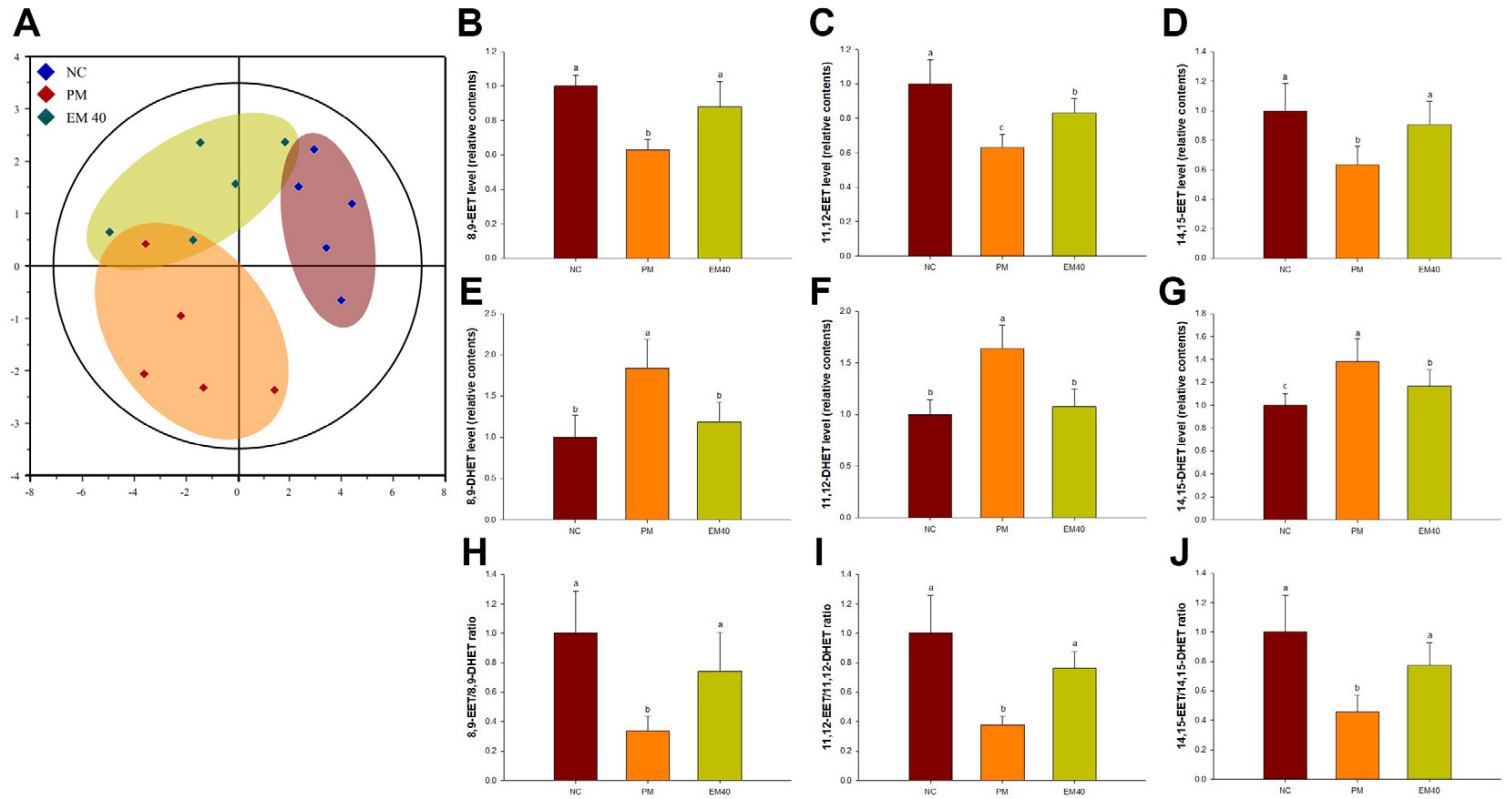
Effect of aqueous extract of matcha (EM) on metabolite changes in cerebral tissue. PLS-DA plot (**A**) and cerebral metabolites (**B-J**). Results shown are mean ± SD (*n* = 5). Data were considered statistically significant at *p* < 0.05 and different small letters represent statistical differences.

Cerebral metabolites changes are shown in Fig. 7. The cerebral 8,9-epoxyeicosatrienoic acid (EET)/8,9-dihydroxyeicosatrienoic acid (DHET) (0.34), 11,12-EET/11,12-DHET (0.38), and 14,15-EET/14,15-DHET (0.46) ratios of the PM group were reduced compared to that of the NC group. However, the cerebral 8,9-EET/8,9-DHET (0.74), 11,12-EET/11,12-DHET (0.76), and 14,15-EET/14,15-DHET (0.77) ratios of the EM40 group were increased.

### Metabolome Aanalysis

**Fig. 8:**
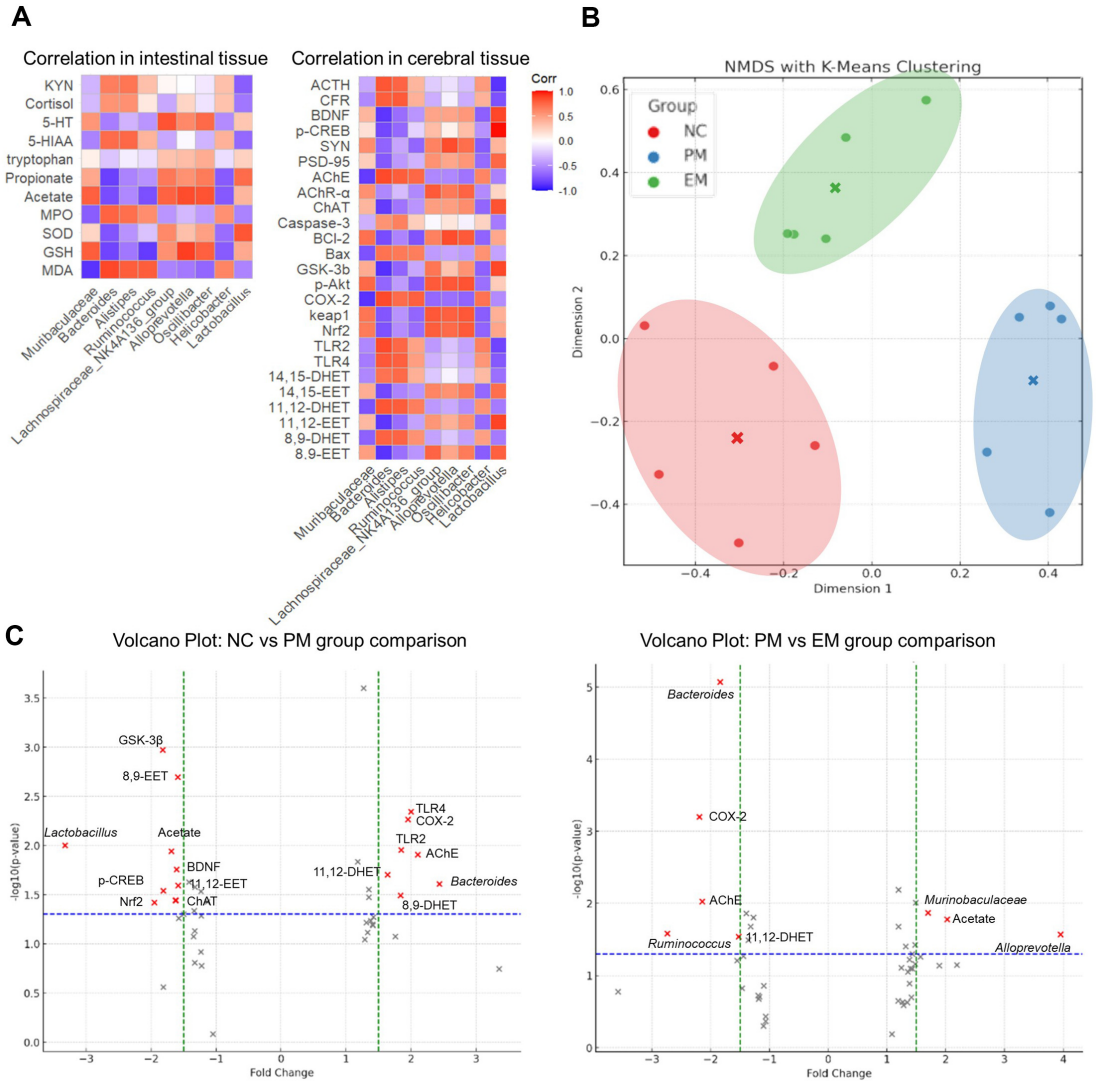
Effect of aqueous extract of matcha (EM) on metabolite analysis. Heat map of correlation analysis in intestinal and cerebral tissues (**A**) NMDS clustering (**B**) volcano pot of NC and PM group comparison (**C**) and volcano pot of PM vs EM group comparison (**D**).

Correlation analysis in intestinal and cerebral tissues were presented in Fig. 8A. A comparative analysis was conducted using the changes in microorganisms detected in the intestines and the analyzed indicators. In addition, it was confirmed that NMDS cluster and volcano plot were formed using the analyzed indicators in Fig. 8B and 8C. The clustering results indicate the structural characteristics of each group, and the relationships between clusters and consistency within each group were confirmed. The indicators, including GSK-3β, 8,9-EET, 8,9-DHET, 11,12-EET, 11,12-DHET, *Lactobacillus*, *Bacteroides*, intestinal acetate, p-CREB, BDNF, Nrf2, ChAT, TLR4, TLR2, and COX-2, showed a significantly changes between NC and PM groups. The indicators, including *Bacteroides*, *Ruminococcus*, *Murinobaculaceae*, *Allopreyotella*, COX-2, AChE, 11,12-DHET, intestinal acetate, showed a significantly changes between PM and EM groups.

## Discussion

PM_2.5_ is an environmental component of air pollution that causes various diseases [1]. PM_2.5_ is normally absorbed into the nasal cavity, lungs, and skin tissues, and leads to systemic inflammation and cytotoxicity [3]. In addition, PM_2.5_ absorbed into the GI tract might induce intestinal dysfunction and the production of oxidative stress and inflammation response and cause TJ protein damage and dysbiosis [5]. Abnormal changes in gut health cause damage to various intestinal metabolic pathways, such as damage to GBA, which is involved in hormonal regulation [2]. Accordingly, although studies on intestinal health damage due to PM_2.5_ exposure are continuously reported, the specific mechanism of green tea is not clear. Therefore, this study evaluated the protective effect of green tea as matcha against chronic PM_2.5_-induced GBA dysfunction.

The oxidative stress and inflammatory response generated by PM_2.5_ are essential in developing intestinal dysfunction [7]. PM_2.5_ is generally exposed through skin tissues and/or the respiratory airway, but it was recently reported that PM_2.5_ entered through the GI tract [3, 5]. The absorbed PM_2.5_ can lead to intestinal damage by generating cytokines and oxidative stresses [18]. In addition, PM_2.5_ contains various heavy metals such as Fe^3+^, Mg^2+^, Cu^2+^, Co^3+^, Cd^2+^, and Pb^2+^, which induce oxidative stresses and radicals [19]. These heavy metals cause cytotoxicity and the imbalance of antioxidant systems in various organs, including the brain, liver, lungs, and colon [1]. Thus, PM_2.5_ and its derivates can be eliminated and scavenged through the intestinal antioxidant scavenging system, but those lead to excessive oxidative stress, which damages the gut barrier [8]. Therefore, antioxidant functions, including MDA production, reduced GSH levels, SOD activities, and MPO activities, and inflammatory markers, including inflammatory cytokines, were investigated in intestinal tissues and serum to assess the protective effects of EM in intestinal tissue (Fig. 1). This antioxidant system protection effect is believed to be due to the excellent antioxidant activity of green tea itself, and plays a role in sufficiently eliminating the toxicity of various heavy metals [10]. In a previous study, PM_2.5_ was found to cause the dysfunction of the antioxidant system in dermal, pulmonary, and cerebral tissues, and the intake of EM regulated SOD activity and reduced GSH levels and lipid peroxide [16]. Also, green tea polyphenols reduced apoptosis with the inhibition of MDA production and reduction of SOD activity in PM_2.5_-treated A549 cells [20]. The administration of EM protected the hepatic and cerebral antioxidant system by regulating the production of MDA and SOD activity in diabetic mice [10]. In addition, green tea extract lowered pro-inflammatory genes such as iNOS, MCP-1, and MPO by regulation of NF-κB phosphorylation to protect against high-fat diet-induced nonalcoholic steatohepatitis [2]. Therefore, EM significantly prevented intestinal antioxidant damage and serum inflammatory indicators, which can stimulate systemic inflammation in the whole body induced by PM_2.5_ toxicity. It might help improve gut health and be used as an industrial material with antioxidant activity.

The gut microbiome sustains a vital metabolic system related to energy metabolism, immunological defense system, maintenance of hormonal function, and digestion [21]. However, the disturbance of the gut microbiota is related to the risk of developing an inflammatory response, imbalance of nutrition metabolism, allergic diseases, and ultimately brain damage [9]. It has also been suggested that chronic exposure to environmental xenobiotic toxicants is significantly associated with factors that shape or change the gut microbiota [8]. PM_2.5_ and inflammatory cytokines increase the growth of gram-negative bacteria and stimulate the secretion of lipopolysaccharide (LPS) in the outer membrane of gram-negative bacteria [18]. In this study, the changes at the Phylum level in the PM group were no different from those in the NC group. However, there were significant changes in various microbiomes at the Genus level. In addition, EM showed regulatory effects against PM_2.5_-induced dysbiosis by changing the diverse microbiota in feces (Fig. 2). The administration of EM increased the abundance of *Lactobacilli*, *Oscillibacter*, *Lachnospiraceae* NK4A136 group, *Alloprevotella*, and *Muribaculaceae*. *Lactobacilli* scavenges superoxide and hydroxyl free radicals and regulates the differentiation of T cells, producing the IL-22 cytokine [22]. *Oscillibacter* can up-regulate the intestinal expression of TJ protein and inhibit the absorption and accumulation of Pb [23], and provide energy to intestinal cells to produce butyrate, which plays an important role in maintaining gut homeostasis and enhancing the biosynthesis of 5-HT [24]. The *Lachnospiraceae* NK4A136 group and *Alloprevotella* are known to improve the gut barrier function as potential butyrate producers [23]. *Muribaculaceae* reduce TNF-α and IL-6, and increase IL-10 [6], and ferment nondigestible carbohydrates to SCFA such as butyrate, propionate, and acetate [25]. In addition, the intake of EM reduced the abundance of *Ruminococcaceae* promoting the production of cytokines such as IL-1β, IL-2, and IL-6 [26], *Helicobacter* inducing chronic inflammation and gastric cancer [27], and *Alistipes* causing serotonergic imbalance, anxiety and depression [28]. It has been reported that these microbial changes are regulated by various green tea catechins including EGCG, and that *Lactobacillus* and *Muribaculaceae* utilize catechins to produce SCFAs, which lower the intestinal pH and improve the intestinal environment [29, 30]. Similar to previous studies, PM_2.5_ induced a decrease in intestinal SCFAs, and up-regulated serum inflammatory cytokines (Fig. 1), but the intake of EM suppressed these abnormal changes by regulating the intestinal environment. Therefore, the supplement EM can be used as prebiotics with anti-inflammatory and gut-barrier function improvement effects as an SCFA producer. However, the microbial changes associated with the changes in the EM 40 group at the phylum level should be further confirmed.

Environmental exposure to PM_2.5_ that enters the GI tract can cause intestinal inflammation and cytotoxicity [5]. PM_2.5_ contains a variety of toxicants, such as heavy metals, carbon monoxide, and sulfur dioxide [7]. Exposure to PM_2.5_ causes gut barrier dysfunction by decreasing the expression of TJ proteins, including claudin-1, occludin, and ZO-1 [5]. This disruption of the intestinal barrier induces the permeation of toxic molecules, including pathogens, toxins, cytokines, and antigens [8]. In addition, damage to the intestinal barrier affects the supply of nutrients to the body by inhibiting energy and vital metabolism [21]. Therefore, modifying intestinal barrier function and structure induced by the chronic and continuous formation of oxidative stress and cytotoxicity is related to the pathogenesis of intestinal diseases and systemic inflammation [5]. In addition, the toxicants in PM_2.5_ are strong oxidative compounds that produce oxidative stresses and inflammatory cytokines in gut tissue [19, 21]. Furthermore, PM_2.5_ stimulates the growth of gram-negative bacteria, producing LPS, which can combine with TLR4 [18]. The activated TLR4 triggers the phosphorylation of JNK through the mitogen-activated protein kinase (MAPK) pathway and nuclear factor kappa-light-chain-enhancer of the activated B cell (NF-κB) pathway [31]. Inflammatory cytokines such as IL-1β, IL-6, and TNF-α were produced in intestinal tissue through both inflammatory pathways [32]. Phosphorylated JNK also induces a caspase cascade by increasing the BAX activation and the cytochrome c release [31]. Thus, PM_2.5_ ultimately indicates an abnormal gut microenvironment through inflammatory reaction and caspase cascade [18]. EGCG ameliorated intestinal dysfunction and barrier deficit by restoring the expression level of ZO-1, occludin, and claudin-1 and activating antioxidant enzymes such as catalase, SOD, and glutathione peroxidase [33]. Additionally, the increase in *Alistipes* and *Helicobacter* as gram-negative bacteria confirmed in this study was reported to accelerate disruption of the intestinal barrier by stimulating the release of LPS and inflammatory cytokines [27, 32], whereas the administration of EM significantly regulated dysbiosis. In addition, black tea inhibited inflammatory cytokines including IL-12, IL-23, IL-6, and IL-1β, and suppressed IκBα phosphorylation and activity of NF-κB in an LPS-induced colitis model [34]. Gallic acid as the phenolic acid of green tea played a protective and anti-inflammatory role by regulating expressions of IL-21, IL-23 and Nrf2 in DSS-induced BALB/c mice [35]. L-theanine suppressed the expression levels of TNF-α, IL-1β, IL-6, iNOS, and COX-2 in DSS-induced colitis, and relieved phosphorylation of GSK-3β and Akt/mTOR in Cd-induced ICR mice [36]. In this study, the administration of EM can protect against gut damage by regulating inflammation and caspase-induced cytotoxicity as prebiotics containing various physiological compounds. Moreover, it is suggested that changes in the gut microbiome with the intake of EM can help maintain gut health. EM up-regulated changes of various bacteria such as *Lactobacilli*, *Lachnospiraceae* NK4A136 group, and *Alloprevotella*. The increase in *Lactobacilli* suppressed the secretion of inflammatory cytokines such as TNF-α, IL-6, IL-1β, and IL-10 [22]. Furthermore, the *Lachnospiraceae* NK4A136 group as a potential butyrate producer presented an anti-inflammatory effect by suppressing inflammatory indicators such as TNF-α, IL-1β, IL-6, and iNOS in gut tissues [37]. Thus, EM might have significant anti-inflammatory effects through interaction with intestinal microbiota. This protective effect of EM ultimately helps reduce inflammatory response and toxicity in brain tissue (Fig. 5). In particular, the increased inflammatory response induced by PM_2.5_ oxidizes EETs, a type of arachidonic acid, through the catalytic reaction of soluble epoxide hydrolase (sEH) [38]. The oxidation of EETs, a form of DHETs, promotes the inflammatory response by up-regulating COX-2 and NF-κB [39]. This mechanism continuously increases nitric oxide production and iNOS expression, accelerating the inflammatory response and promoting neuronal death in the brain. Similar to these results, exposure to PM_2.5_ stimulated EETs into DHET in brain tissue (Fig. 7). However, uptake of EM might have helped suppress the continued expression of inflammation by inhibiting the oxidation of EETs. Therefore, ingestion of EM may help suppress inflammatory responses by suppressing abnormal metabolite changes in arachidonic acid. In conclusion, EM might be used for a potential functional food material with a considerable protective effect by regulating the GBA function through intestinal inflammatory response, cytotoxicity, and TJ barrier function.

Tryptophan produced by gut microbiota is important to balance the whole body [40]. In particular, tryptamine catabolized from tryptophan stimulates the release of serotonin (5-HT) by enterochromaffin cells [41]. The released 5-HT promotes gastrointestinal motility by accelerating the enteric neuronal system and continuously helps absorb 5-HT into the bloodstream [42]. However, along with serotonergic metabolism, tryptophan is catalyzed to QUIN as a systemic toxic transmitter that induces the excessive stimulation of the N-methyl-D-aspartate (NMDA) receptor, which produces central cholinergic neurotoxicity [2]. In particular, the NMDA receptor activated by QUIN down-regulates synaptic activity, which is related to cognitive and memory function, by regulating the expression of ChAT in the cerebral cortex and amygdala [16]. Similar to this result, PM_2.5_ was confirmed to induce the impairment of cholinergic and synaptic function in brain tissue (Fig. 6). In the case of PM_2.5_ with *Ruminococcaceae* and *Alistipes* producing indole compounds, tryptophan is transferred to L-kynurenine and QUIN as a metabolite of the tryptophan used in the production of niacin [40]. However, the over-production of L-kynurenine and QUIN inhibits the transformation of tryptophan to 5-HT and leads to an additional imbalance of serotonergic metabolism [41]. The green tea catechin EGCG prevented QUIN-induced apoptotic signals via the PI3K pathway [42]. Thus, continuous exposure to PM_2.5_ might cause an imbalance in hormonal regulation metabolism and ultimately lead to damage to brain tissue through GBA dysfunction. On the other hand, the regulation of the gut microbiome with the administration of EM also plays an important role in affecting serotonergic balance. *Alistipes* increased by PM_2.5_ exposure is known to produce tryptophanase, which inhibits serotonin biosynthesis in the colon [2]. However, treatment with EM significantly down-regulated *Alistipes* in the fecal microbiome (Fig. 2). In addition, *Lactobacillus* increased through intake of EM is reported to stimulate the production of serotonin, acetylcholine (ACh), and γ-aminobutyric acid (GABA) as neurotransmitters [43]. In particular, the green tea amino acid L-theanine is easily transferred to 5-HT, dopamine, and GABA by the microbiome [44]. Also, the increased absorption of 5-HT can inhibit the production of QUIN and cortisol levels in serum [40]. Therefore, these results suggest that consumption of EM might alleviate hormonal dysfunction by improving intestinal tryptophan metabolism and ultimately help improve PM_2.5_-induced synaptic dysfunction. Further research about the additional and detailed mechanism is needed, but EM containing physiological compounds might support beneficial changes in tryptophan metabolites for brain function.

These results were derived from the various catechins present in EM. The contents of the significant catechin components, including EGC, EC, EGCG, and ECG, of EM used in this study were shown to be higher than those of leaf green tea extract through a previous study [10]. Specifically, the EGC in EM was 49.49 mg/g, which was 1.26 times higher than that of leaf green tea extract, EC was 8.74 mg/g, which was 1.67 times higher, EGCG was 50.24 mg/g, which was 1.32 times higher, and ECG was 13.75 mg/g, which was 1.30 times higher [10]. This high catechin content suggests that matcha may provide stronger antioxidant and anti-inflammatory effects than leaf green tea and supports the possibility that it may be more effective in alleviating gut-brain axis dysfunction caused by PM_2.5_.

## Conclusion

In summary, EM suppressed the abnormal intestinal system and microenvironment change induced by PM_2.5_. The supplement of EM regulated tryptophan metabolism and hormonal changes. EM presented the ameliorating effect against cerebral damage through this change, including cerebral inflammation, cytotoxicity, synaptic damage, and arachidonic metabolite changes. Taken together, this results suggests that green tea as matcha might be useful as a considerable material for functional foods, pharmaceutics, and nutraceuticals with an ameliorating effect against PM_2.5_-induced brain dysfunction through regulation of intestinal inflammatory response, hormonal change, and tight junction function.

## Supplemental Materials

Supplementary data for this paper are available on-line only at http://jmb.or.kr.
