# Supplementary material for: Green Tea Attenuates the Particulate Matter (PM)2.5-Exposed Gut-Brain Axis Dysfunction through Regulation of Intestinal Microenvironment and Hormonal Changes
Source: J Microbiol Biotechnol. 2024 Oct 31;34(12):2492–505. doi: 10.4014/jmb.2409.09035 (PMC11729334; doi:10.4014/jmb.2409.09035)
Supplement: Supplementary file 1 [file jmb-34-12-2492-supple.pdf]

J. Microbiol. Biotechnol. 2024. 34(12): 2492–2505
https://doi.org/10.4014/jmb.2409.09035

Supplementary Tables

Table S1. List of antibody details used in this study.

Antibody	Catalog	Concentration	Manufacture
β-actin	sc-69879	1:1,000	Santa Cruz Biotech. (Dallas, TX, USA)
TLR4	sc-52962	1:1,000	Santa Cruz Biotech. (Dallas, TX, USA)
TLR2	sc-21759	1:1,000	Santa Cruz Biotech. (Dallas, TX, USA)
p-JNK	sc-6254	1:1,000	Santa Cruz Biotech. (Dallas, TX, USA)
p-IκB-α	sc-8404	1:1,000	Santa Cruz Biotech. (Dallas, TX, USA)
COX-2	sc-376861	1:1,000	Santa Cruz Biotech. (Dallas, TX, USA)
iNOS	sc-7271	1:1,000	Santa Cruz Biotech. (Dallas, TX, USA)
Caspase-1	sc-392736	1:1,000	Santa Cruz Biotech. (Dallas, TX, USA)
IL-1β	sc-4592	1:1,000	Santa Cruz Biotech. (Dallas, TX, USA)
BAX	sc-7480	1:1,000	Santa Cruz Biotech. (Dallas, TX, USA)
ZO-1	sc-33725	1:1,000	Santa Cruz Biotech. (Dallas, TX, USA)
Occludin	sc-133256	1:1,000	Santa Cruz Biotech. (Dallas, TX, USA)
Claudin-1	sc-166338	1:1,000	Santa Cruz Biotech. (Dallas, TX, USA)
MUC2	sc-515032	1:1,000	Santa Cruz Biotech. (Dallas, TX, USA)
IDO-1	sc-137012	1:1,000	Santa Cruz Biotech. (Dallas, TX, USA)
Nrf2	sc-365949	1:1,000	Santa Cruz Biotech. (Dallas, TX, USA)
keap1	sc-365626	1:1,000	Santa Cruz Biotech. (Dallas, TX, USA)
COX-2	sc-376861	1:1,000	Santa Cruz Biotech. (Dallas, TX, USA)
p-Akt	sc-393887	1:1,000	Santa Cruz Biotech. (Dallas, TX, USA)
p-GSK-3β	sc-373800	1:1,000	Santa Cruz Biotech. (Dallas, TX, USA)
BCl-2	sc-509	1:1,000	Santa Cruz Biotech. (Dallas, TX, USA)
HO-1	sc-136960	1:1,000	Santa Cruz Biotech. (Dallas, TX, USA)
AChR-α3	sc-365479	1:1,000	Santa Cruz Biotech. (Dallas, TX, USA)

AChE	sc-373901	1:1,000	Santa Cruz Biotech. (Dallas, TX, USA)
PSD-95	sc-32290	1:1,000	Santa Cruz Biotech. (Dallas, TX, USA)
SYN	sc-17750	1:1,000	Santa Cruz Biotech. (Dallas, TX, USA)
p-CREB	sc-81486	1:1,000	Santa Cruz Biotech. (Dallas, TX, USA)
CRF	sc-293187	1:1,000	Santa Cruz Biotech. (Dallas, TX, USA)
ACTH	sc-57018	1:1,000	Santa Cruz Biotech. (Dallas, TX, USA)
Caspase-7	CSB-PA15479A0Rb	1:1,000	Cusabio (Hubei, China)
Caspase-3	CSB-PA05689A0Rb	1:1,000	Cusabio (Hubei, China)
ChAT	20747-1AP	1:1,000	Bioneer (Daejeon, Korea)
p-NF-κB	3033S	1:1,000	Cell Signaling Tech. (Danvers, MA, USA)
TNF-α	5178SC	1:1,000	Cell Signaling Tech. (Danvers, MA, USA)
BDNF	16696S	1:1,000	Cell Signaling Tech. (Danvers, MA, USA)
Secondary anti-rabbit	7074S	1:5,000	Cell Signaling Tech. (Danvers, MA, USA)
Secondary anti-mouse	7076S	1:5,000	Cell Signaling Tech. (Danvers, MA, USA)

Table S2. Retention time, MRM transitions and collision energy of the analytes.

Analytes	RT[1] (min)	Precursor ion (m/z)	Product ion (m/z)	CE[2] (eV)
Quinolinic acid (QUIN)	1.03	168.10	151.10	20
Tryptophane	2.84	205.00	188.00	40
5-hydroxyindoleacetic acid (5-HIAA)	3.06	192.00	146.20	10
5-hydroxytryptamine (5-HT; Serotonin)	4.04	177.01	115.00	40
Cortisol	4.42	363.20	121.10	20
8,9-epoxyeicosatrienoic acid (EET)	4.40	319.20	167.00	20
8,9-dihydroxyeicosatrienoic acid (DHET)	3.78	337.20	127.00	40
11,12-EET	4.35	319.20	167.00	20
11,12-DHET	3.65	337.20	167.00	25
14,15-EET	4.25	319.20	219.20	20
14,15-DHET	3.60	337.10	207.20	25

[1] RT: Retention time; [2] CE: Collision energy

Table S3. Catechin contents of aqueous extract of leaf green tea and matcha green tea.[1)]

	EGC	EC	EGCG	ECG	Total catechin
Leaf green tea	39.27±0.58[b]	5.22±0.21[b]	38.18±0.08[b]	10.57±0.07[b]	93.24±0.33[b]
Matcha green tea	49.49±0.12[a]	8.74±0.06[a]	50.24±0.57[a]	13.75±0.11[a]	122.22±0.21[a]

Results are mean±SD (n=3). Data were statistically represented at $p<0.05$, and different small alphabets mean statistical significance.

[1)] Kim, J. M., Lee, U., Kang, J. Y., Park, S. K., Shin, E. J., Moon, J. H., & Heo, H. J. (2021). Protective effect of matcha green tea (*Camellia sinensis*) extract on high glucose-and oleic acid-induced hepatic inflammatory effect. *Korean Journal of Food Science and Technology*, *53*(3), 267-277. https://doi:10.9721/KJFST.2021.53.3.267.

Table S4. Catechin contents of aqueous extract of leaf green tea and matcha green tea.[1)]

	TPC[1]	TFC[2]	ABTS[3]	DPPH[4]	MDA[5]
Leaf green tea	235.92±1.25[b]	83.87±1.03[b]	310.48±2.48[a]	380.98±5.15[a]	68.15±3.57[a]
Matcha green tea	325.00±7.35[a]	104.24±5.17[a]	276.56±4.25[b]	321.05±3.24[b]	59.98±2.48[b]

[1]TPC, total phenolic content; [2]TFC, total flavonoid content; [3]ABTS, ABTS radical scavenging activity; [4]DPPH, DPPH radical scavenging activity; [5]MDA, malondialdehyde (MDA) inhibitory effect. Results shown are mean±SD (n=3). Data were statistically represented at $p<0.05$, and different small alphabets mean statistical significance. Results of TPC and TFC are presented as mg of GAE/g and mg of RE/g, respectively. Results of ABTS, DPPH and MDA are presented as IC_{50} value (μg/mL).

[1)] Kim, J. M., Lee, U., Kang, J. Y., Park, S. K., Shin, E. J., Moon, J. H., & Heo, H. J. (2021). Protective effect of matcha green tea (*Camellia sinensis*) extract on high glucose-and oleic acid-induced hepatic inflammatory effect. *Korean Journal of Food Science and Technology*, *53*(3), 267-277. https://doi:10.9721/KJFST.2021.53.3.267.
